# Functional Feeds to Tackle Meagre (*Argyrosomus regius*) Stress: Physiological Responses under Acute Stressful Handling Conditions

**DOI:** 10.3390/md19110598

**Published:** 2021-10-21

**Authors:** Marta Monteiro, Carla Sousa, Filipe Coutinho, Carolina Castro, Filipa Fontinha, Inês Guerreiro, Pedro Pousão, Elisabete Matos, Patrícia Díaz-Rosales, Aires Oliva-Teles, Paula Enes, Ana Couto

**Affiliations:** 1 Interdisciplinary Centre of Marine and Environmental Research (CIIMAR), University of Porto, Terminal de Cruzeiros do Porto de Leixões, Av. General Norton de Matos, s/n, 4450-208 Matosinhos, Portugal; flipcoutinho@gmail.com (F.C.); carolinacastro23@gmail.com (C.C.); filipafontinha@hotmail.com (F.F.); imsguerreiro@gmail.com (I.G.); pdiazrosales@gmail.com (P.D.-R.); enes.ciimar@gmail.com (P.E.); acouto@fc.up.pt (A.C.); 2 Departament of Biology, Faculty of Ciências, University of Porto, Rua do Campo Alegre, Building FC4, 4169-007 Porto, Portugal; carladaniela.sousa@gmail.com; 3 Portuguese Institute for Sea and Atmosphere (IPMA), Olhão Pilot Aquaculture Station, Av. 5 de Outubro, s/n, 8700-305 Olhão, Portugal; pedro.pousao@ipma.pt; 4 SORGAL, Oils and Feedd Society, S.A., Estrada Nacional 109 Lugar da Pardala, 3880-728 São João de Ovar, Portugal; ematos@b2e.pt; 5 B2E Blue Bioeconomy CoLab—Colabrtive Laboratory, Av. Liberdade, s/n, 4450-718 Leça da Palmeira, Portugal; 6 Fish Immunology and Patology Group, Center for Animal Health Research (CISA, INIA), Carretera de Algete a El Casar, s/n, 28130 Madrid, Spain

**Keywords:** functional diet, algal extracts, oxidative stress, immunomodulation, aquaculture fish

## Abstract

Marine algae are recognised sources of bioactive compounds that have attracted great interest as nutritional supplements for aquaculture fish. Intensive rearing conditions often expose fish to husbandry-related stressors, rendering fish more susceptible to disease and reducing production yields. The present work evaluated the potential of two marine algae extracts (*Fucus vesiculosus* and *Nannochloropsis gaditana*) as nutritional supplements to mitigate stress effects in meagre (*Argyrosomus regius*) exposed to an acute handling stress (AS). A plant-based diet was used as a control, and three other diets were prepared, which were similar to the control diet but supplemented with 1% of each algal extract or a combination of the two extracts (0.5% each). The effects of supplemented diets on stress biomarkers, antioxidant enzyme activities, and immune response were analysed in fish exposed to AS after 4 weeks of feeding. Supplemented diets did not affect growth performance but the inclusion of *F. vesiculosus* promoted higher feed efficiency, as compared to the control group. Dietary algal extracts supplementation reduced plasma glucose levels, increased white blood cell counts, and reduced the expression of pro-inflammatory genes when compared with the control. *N. gaditana* supplementation led to a reduction in hepatic antioxidant enzyme activity and glutathione levels, while *F. vesiculosus* supplementation increased muscle glutathione reductase activity and reduced lipid peroxidation. These findings support the potential of algal extracts as nutraceuticals in aquafeeds to enhance the ability of fish to cope with husbandry-related stressful conditions and ultimately improve fish health and welfare.

## 1. Introduction

Marine alga-derived molecules have been a focal point for research due to their immunotherapeutic potential to several organisms, including fish. In fact, these were shown to have an active role in cell protection against oxidative damage [1] in the enhancement of fish immune responses [2,3], and have exhibited antiviral and antibacterial properties [3,4,5], which makes them promising functional ingredients of prophylactic agents. Microalgae are well-known sources of high-quality proteins, pigments, long-chain polyunsaturated fatty acids (LC-PUFA), vitamins, polyphenols, flavonoids, and other bioactive substances [6]. On the other hand, macroalgae are sources of proteins, polysaccharides, phenolic compounds, and carotenoids [7,8]. In fish, dietary supplementation with *Nannochloropsis gaditana* promoted growth performance [9] and enhanced immune responses [10,11]. Similarly, dietary supplementation with *Fucus vesiculosus* improved fish immune and antioxidant responses and resistance against pathogens [12,13], and also exhibited genoprotective properties [14,15]. Physiological stress is a major concern in aquaculture production, as it negatively impacts the welfare and overall production yield of fish [16]. Under intensive aquaculture conditions, farmed fish are weakened by several acute and chronic stress conditions, such as crowding, water quality, temperature fluctuation, handling, transportation, or confinement, thus becoming more susceptible to diseases [17,18]. Stress mitigation and increased disease resistance have become a priority for the aquaculture industry, since diseases are responsible for heavy economic losses due to mass mortality, poor product quality, and the costs associated with chemotherapy. One strategy relies on the use of natural sources of substances with therapeutic interest that mitigate stress-negative effects and boost host defence mechanisms, thus preventing farmed fish diseases [19]. With this desideratum, the search for algae’s secondary metabolites with immunotherapeutic properties has gained increased attention [6,20].

Until now, most studies conducted with fish tested whole algae rather than algal extracts [12,21,22]. This can compromise the potential efficiency of bioactive compounds as algae have rigid cell walls that are hard to digest, particularly by carnivorous fish, therefore limiting the availability of several active compounds. The use of algal extracts offers the possibility to directly convey the active compounds, thus overcoming the mentioned problems [23].

Meagre (*Argyrosomus regius*), the model used in the present work, is an emerging fish species in Mediterranean aquaculture, which has been dominated by European seabass (*Dicentrarchus labrax*) and gilthead seabream (*Sparus aurata*) production, thus leading to market saturation and the depreciation of economic value [24]. Meagre is a carnivorous, fast-growing species that presents itself as a promising candidate for aqua-culture diversification due to its high organoleptic characteristics and good market acceptance [25,26]. However, due to its recent cultivation history, it is still sub-adapted to captivity and, therefore, presents stress-related issues.

The present work aimed at evaluating extracts from *N. gaditana* and *F. vesiculosus* as nutritional supplements in aquafeeds for meagre that are capable of alleviating stress-induced negative effects on fish health and welfare. These extracts were selected based on their previously demonstrated antioxidant potential [23] and were incorporated in balanced diets for meagre in order to potentially reduce the acute physiological stress that results from grading/sampling.

## 2. Results

Fish promptly accepted the experimental diets and no significant mortality was recorded between the groups. During the 4-week experimental period, feed intake and fish growth were not affected by dietary treatment, but FE was lower with the diet that included NG, and higher with the diet that included FV (Table 1).

### 2.1. Plasma Metabolite

Compared to unstressed fish (NS), AS increased plasma glucose and cortisol levels (Table 2). Dietary supplementation with FV extract lowered plasmatic glucose levels in both the NS and AS groups, while supplementation with NG extract lowered glucose levels only in the NS group. Plasma lactate was lower in AS than in NS fish, regardless of dietary treatment. Moreover, dietary supplementation with either FV or NG extracts decreased plasma lactate levels, but such an effect was not observed with the combined supplementation of the two extracts.

### 2.2. Hematologic Parameters

The RBC count was higher in AS than in NS fish (Table 3). Similarly, thrombocytes, monocytes, and neutrophils were higher, while lymphocytes were lower in AS fish. Regardless of stress conditions, neutrophils increased with the combined supplementation of FV and NG extracts. Diet supplemented with FV extract increased overall WBC count, while NG extract decreased hematocrit in NS fish. MCV was not affected by dietary treatment or stress conditions.

### 2.3. Gene Expression

**Figure 1:**
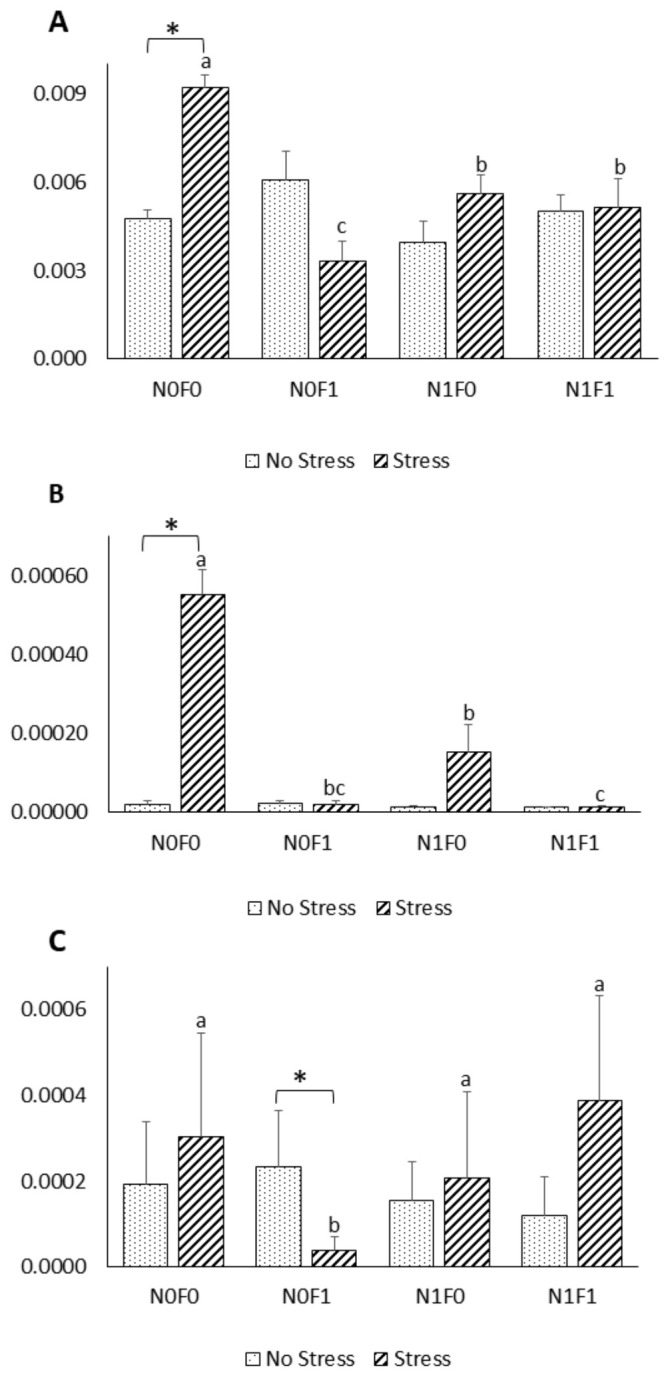
Expression of immune-related genes in the head-kidney of meagre under no-stress (NS) and acute-handling-stress (AS) conditions, after four weeks of feeding with the experimental diets. (**A**) TNF-α, (**B**) IL-1β and (**C**) IL-10. Data are presented as mean ± SD (*n* = 9). Different letters indicate significant differences between dietary treatments ((**A**) *p* = 0.004; (**B**) *p* = 0.001; (**C**) *p* = 0.006). * indicate differences between NS and AS fish within each dietary treatment ((**A**) *p* = 0.035; (**B**) *p* = 0.002; (**C**) *p* = 0.001).

Compared to the NS groups, AS did not affect TNF-α and IL-1β expression in fish fed the extract-supplemented diets, while in the control groups, both genes were upregulated under AS (Figure 1). Further, dietary algal extract supplementation led to a decreased expression of TNF-α and IL-1β under AS. IL-10 expression was only affected by dietary supplementation with FV extract. With this diet, IL-10 was downregulated in AS fish, in comparison to both the NS group and to groups fed the other diets.

**Figure 2:**
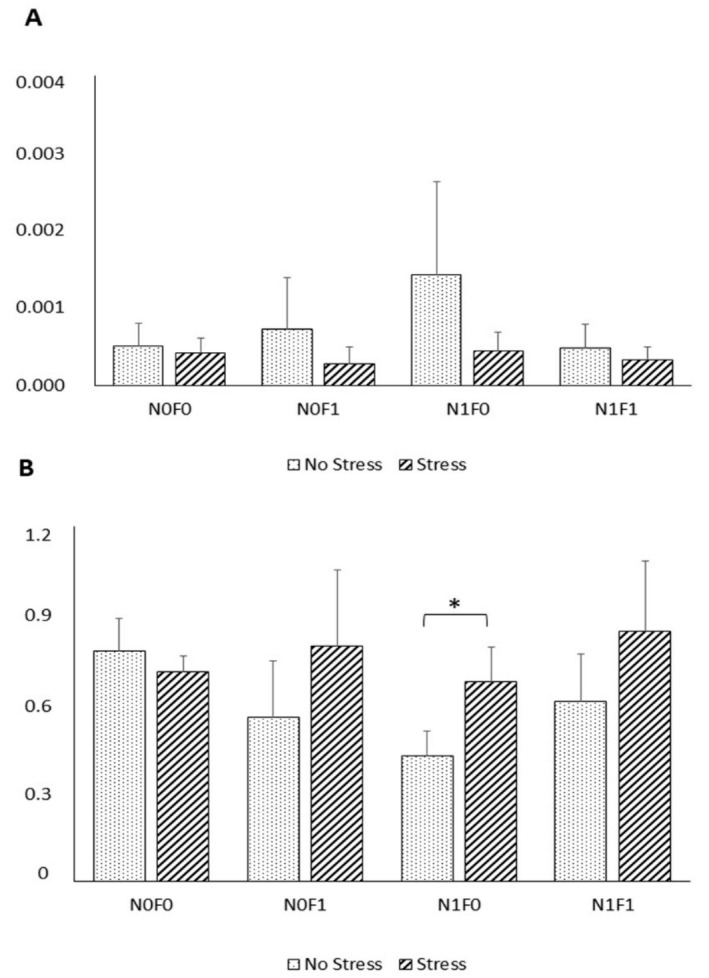
Expression of HSP70 (**A**) and HSP90 (**B**) genes in the muscle of meagre under no-stress (NS) and acute-handling-stress (AS) conditions, after four weeks of feeding with the experimental diets. Data are presented as mean ± SD (*n* = 9). * indicate differences between NS and AS fish within the dietary treatment (*p* = 0.016).

HSP70 and HSP90 expression were not affected by stress or dietary treatment, except in fish fed the diet with NG supplementation, which showed an upregulation of HSP90 under AS (Figure 2).

### 2.4. Oxidative Stress

AS increased the activity of hepatic GPX, GR, and G6PDH. Hepatic SOD, GR, and G6PDH activities (Table 4) were lower in fish fed the diets supplemented with NG extract regardless of stress condition. Hepatic oxidative enzyme activity was not affected by FV extract, except in AS fish, which showed decreased SOD activity.

Hepatic GSSG, OSI, and LPO were not affected by diet composition or stress conditions (Table 5). GSH levels were also not affected by diet composition but were higher in fish fed the NS extract. tGSH decreased in AS fish when fed diets with NG extract supplementation, compared to both NS fish and AS fish fed the other diets.

Muscle SOD activity was not affected by diet composition or stress conditions (Table 6). Dietary supplementation with NG extract increased CAT activity and decreased G6PDH activity. Fish submitted to AS presented increased CAT activity and decreased GPX activity. In NS fish, but not in AS fish, diet FV supplementation led to higher GR activity compared to fish fed diets without supplementation. Dietary supplementation with FV alone resulted in higher GR activity, while dietary supplementation with FV and NG did not affect GR, irrespective of stress conditions. On the other hand, compared to the NS condition, GR activity decreased under AS in fish fed with the FV-supplemented diet.

Muscle GSH was not affected by diet or stress conditions (Table 7). Compared to the NS fish, muscle tGSH, GSSG, OSI, and LPO were higher in fish submitted to AS. Additionally, dietary supplementation with FV resulted in decreased LPO levels.

## 3. Discussion

In fish farming, capture, handling, crowding, confinement, and transport are common practices that affect the stress responses of teleosts [27,28,29]. Over time, these stressors have cumulative and long-term effects on fish [18]. Physiological stress has been reported to have severe negative consequences on growth performance as well as disease resistance [16,30]. Functional feeds constitute a tool to mitigate those effects. In this study, NG and FV extracts were incorporated, as antioxidant additives, in diets for meagre, with the aim of mitigating aquaculture-associated acute handling stress effects.

In fish, increased plasma catecholamines and cortisol are the first systemic response to stress. High cortisol levels induce metabolic processes and lead to increased levels of circulating glucose and lactate. In the present study, these plasma stress indicators varied significantly under the effect of AS, while plasmatic levels of glucose and cortisol increased, and lactate levels decreased, in response to the AS. As for the effect of the dietary treatment, algal extract supplementation did not affect plasma cortisol levels, but glucose and lactate were significantly reduced, particularly in fish fed diets that were supplemented with either isolated NG or FV extracts (N1F0 and N0F1 diets). Under stress, fish are known to increase energy metabolism as a coping mechanism and, since glucose acts as the main energy source, plasma glucose levels are expected to increase as a means of promptly providing the required extra energy [31]. However, in the presence of feed additives of vegetable origin, insulin action has been reported to be enhanced, increasing the uptake of glucose by cells, and decreasing plasma glucose levels in rohu, *Labeo rohita* fingerlings [32] and in rats [33], which may also have occurred in the present work with dietary algal extracts, despite stress conditions. Similarly, a decrease in fish glucose levels caused by *Padina astraulis* methanolic extracts was previously reported in *Mugil cephalus* with supplementation levels as low as 0.5% [34]. As for lactate, higher blood lactate levels occur under stress, as a consequence of increased respiratory activity in the muscle under anaerobic conditions [35,36]. Thus, elevated blood lactate is an indirect indicator of stress, which reflects the imposition of severe exercise in which the tissue requirement for oxygen exceeds the supply [36]. However, in this study, the opposite was verified. Even though the disturbance of blood lactate levels was only moderate (ranging from 2.6 to 3.9 mmol L^−1^), suggesting that the degree of respiratory stress experienced was not excessive, the findings that dietary algal supplementation led to lower lactate levels may reveal a relationship between dietary algal extract supplementation and muscle performance and fatigue. Moreover, previous studies in mammals have linked the nutritional status to fluctuations in levels of lactate under physical stress [37,38]. While nutrient-deficient diets led to higher lactate levels under physical stress, the opposite was verified when a nutritionally balanced diet was supplied [37,38], similarly to the present study, which may partially explain the results obtained herein. Further studies are, however, necessary to better understand the role of dietary algal extracts and their bioactive compounds on lactate metabolism under stressful conditions.

Hematological parameters can be used to monitor the health status of fish in response to changes related to nutrition, water quality, stress, and diseases [39]. The increasing of RBC has been reported as a strategy to enhance the capacity of blood to carry oxygen under a high energy demand condition, such as stress [40,41]. Accordingly, in this study, RBC was increased under stress conditions, but was unaffected by dietary supplementation.

Fish leukocyte modulation under stress conditions follows a typical profile, consisting of decreased numbers of circulating lymphocytes due to their migration from the blood to tissues, and the inhibition of lymphocyte proliferation by stress hormones [42,43]. Additionally, monocyte and neutrophil numbers increase since they are recruited to circulation, with higher numbers of circulating neutrophils being characteristic of acute stress situations [44]. The fish included in this study presented a typical leukogram in response to an acute handling of stress, and dietary extract supplementation did not modulate the leukocyte response. However, the increase in total WBC observed in fish fed with the FV-supplemented diets could be a result of the improved immune response, as shown with other feed additives, such as vitamin C, white button mushroom and nucleotides [45,46,47]. Hence, FV extract supplementation may enhance fish stress and disease resistance under stressful conditions.

Hematocrit variation can be indicative of stress due to changes in blood dilution or concentration that are related to osmoregulatory disorders [48]. In the present study, however, hematocrit was not affected by stress. A reduction in hematocrit was, however, observed in fish fed the diet supplemented with NG, but only in NS fish. Normally, these findings would suggest that NG supplementation presents adverse effects on red blood cell synthesis/destruction or cell volume [49]. In the present study, however, no other hematological parameters were affected by NG supplementation, and thus, the cause of reduced hematocrit cannot be ascertained. Further studies should be conducted on the hematological effects of NG supplementation to discard possible toxicity effects. 

Several studies previously showed that stress can be immunosuppressive, and hence, may be detrimental to fish health [50]. Acute stressors have been reported to either increase or decrease the production of pro-inflammatory cytokines, such as IL-1β and TNF-α, depending on the stimulus applied, and the tissue and species considered [51,52]. These apparently conflicting results may also be related to a delayed stress-induced variation of these pro-inflammatory cytokines, and the lack of a well-established peak response [50]. Cytokines are important signalling molecules of the immune system that intervene in both innate and acquired responses and are modulated by diverse stimuli [53]. While TNF-α and IL-1β are key pro-inflammatory cytokines that are mainly produced by active macrophages, IL-10 acts as an anti-inflammatory cytokine by downregulating the production of pro-inflammatory cytokines [53]. In the present work, expression of the pro-inflammatory genes TNF-α and IL-1β was induced under AS conditions in fish fed the control diet. However, fish fed the extract-supplemented diets were able to keep the pro-inflammatory cytokines’ expression levels close to the basal levels, indicating that the exacerbated inflammatory responses induced by AS can be controlled by the dietary algal extract supplementation. Similar effects were also observed by [54], who showed that lipid extracts from macroalgae inhibited the production of pro-inflammatory cytokines on LPS-stimulated human THP-1 macrophages. Previous reports linked reactive oxygen species (ROS) formation to pro-inflammatory cytokines’ action, specifically that of TNF-α, via their mutual influence within a positive feedback loop [55,56]. In light of the results obtained by [23] and those reported herein (Table 1), dietary NG and FV supplementation could be responsible for keeping cytokine expression levels in AS fish similar to those in NS fish through their direct influence on ROS. Furthermore, phenolic compounds, including terpenoids, anthocyanins and phenolic rich extracts, have been reported to inhibit the nuclear translocation of nuclear factor-κB, which is involved in the activation of several pro-inflammatory genes, but further investigations are needed to unveil the exact mechanisms of action [57,58]. Thus, by preventing an excessive pro-inflammatory response, which in turn, leads to increased susceptibility to secondary infections [59], dietary supplementation of phenolic-rich extracts (FV and NG) could be used as a valuable prophylactic immunotherapy strategy in aquaculture facilities, as suggested by [60].

HSPs are a super-family of highly conserved intracellular proteins found in all organisms that are involved in protein folding and transport, receptor binding, and proteolysis [61]. Several HSPs, including HSP70 and HSP90, were reported to increase in concentration in response to a variety of abiotic stressors [62]. In this study, however, no significant differences were observed in HSP70 expression between NS and AS fish. Fish responses to external stimuli differ greatly between fish species. It is unclear why different fish species exposed to stress have varied responses in terms of HSP expression. A possible explanation could be that the level of constitutive expression of HSP is already high enough to overcome the potentially harmful effects of stressors, such as increased temperature, as long as it remains within the normal physiological range for fish [63]. Similarly, other studies failed to detect differences in HSP expression in fish exposed to common aquaculture stressors, including handling stress [64,65], suggesting that further studies are needed to understand the relation between stressors and HSP expression. 

Nonetheless, compared to the NS group, under AS, a trend towards increased HSP90 expression in fish fed with diet was noticed. Such results suggest that NG inclusion, under stressful conditions, promotes cell protection against damage induced by stress, as HSP90 actively intervenes in the immune, apoptotic, and inflammatory processes [61].

In the present work, fish under AS showed increased hepatic and muscle antioxidant enzyme activity, likely as a response to increased ROS production caused by AS. In an extreme situation, if ROS levels surpass fish antioxidant defences, oxidative stress occurs, ultimately leading to cellular damage [66]. Moreover, the antioxidant enzyme activity was much higher in the liver than in the muscle, reflecting the higher metabolic rate of the liver, and its central role in energy metabolism and detoxification. Indeed, the liver is a central organ for the development of antioxidant defences [67,68]. The current results are in line with previous reports that were based on largemouth bass (*Micropterus salmoides*) and common carp (*Cyprinus carpio*) being exposed to short-term stresses [67,69]. Regarding dietary effect, while FV extract did not affect hepatic enzyme activity, it increased GR activity in the muscle. NG extract decreased SOD and GR activity in the liver and increased CAT activity in the muscle. G6PDH was reduced in both tissues in fish fed with NG-supplemented diets. Contrarily, studies in other species report that functional feed additives, such as marine algae and their bioactive compounds (e.g., β-glucan and fucoidan), increase antioxidant enzyme activity under stressful conditions [1,51,70]. Furthermore, the decreased antioxidant enzyme activity in fish fed the diet containing NG extract could be linked to the extract’s ability to directly scavenge ROS, as previously reported by [23], thereby minimizing the need for antioxidant enzymes to detoxify ROS.

Lipid peroxidation (LPO) is a well-established marker for oxidative tissue damage, and therefore, a reliable indicator of oxidative stress [71]. In the present study, despite variations in oxidative stress enzyme activity, hepatic LPO levels were not affected by stress or diet composition. In contrast, in the muscle, LPO was higher under the AS condition and decreased in fish fed the diet that included FV extract, suggesting a protective role of FV extract against oxidative stress, particularly in relation to stressful events. These results are in line with what was expected since polyphenolic compounds derived from the brown macroalgae methanol extracts, such as the FV extract used herein (Table 1), have been reported to decrease lipid peroxidation [72,73]. The algal extracts used in the present work were optimised with the use of hydro-alcoholic solvents (methanol and ethanol) in previous work [23] and selected based on their phenolic content and antioxidant potential, and therefore, were expected to have an effect on overall performance, antioxidant status, immune responses and disease resistance, as reviewed by [74]. Although no effects were observed in terms of growth performance, antioxidant status and immune and humoral responses were found to be modulated by supplementation with phenolic-rich extracts. However, the exact mechanisms of action of the extract have yet to be understood.

In conclusion, this study showed that meagre that were subjected to acute handling stress exhibited reduced plasma lactate and increased plasma glucose and cortisol levels. AS also induced hepatic expression of pro-inflammatory genes as well as the activity of hepatic and muscle oxidative stress enzymes. On the other hand, dietary supplementation of NG extract reduced oxidative stress responses and FV extract reduced lipid peroxidation, while both extracts reduced inflammatory responses. By reducing potential ROS damage and minimizing the inflammatory response induced by acute handling stress, supplementation with phenolic-rich extracts (FV and NG) could provide valuable immunotherapeutic tools in terms of the responses of fish under stressful conditions.

As NG and FV extracts induce different actions in fish, further studies on dietary supplementation with both extracts should be conducted to determine the possible mechanisms of dietary algae in the alleviation of fish stress. Moreover, future studies should focus on how to take advantage of the beneficial effects of each extract, ultimately providing a valuable resource to protect against stressful conditions.

## 4. Materials and Methods

### 4.1. Algae Extracts’ Obtention

Solvent mixtures and the extraction methodology were selected based on previous results obtained on the antioxidant activity and phenolic content of micro and macroalgae extracts [23]. Algal extracts were prepared from freeze-dried biomass from *F. vesiculosus* (FV) obtained from Alga+ (Ílhavo, Portugal) and *N. gaditana* (NG) obtained from Buggy Power S.L. (San Pedro del Pinatar, Murcia, Spain). Briefly, 67 g of freeze-dried algal biomass was milled and then mixed with 1.3 L of ethanol/water (80:20 v/v; for NG) or methanol/water (50:50 v/v; for FV). The solvent mixtures and the mixtures were then vortexed and incubated for 30 min with continuous orbital agitation, in the dark, at room temperature. Each mixture was centrifuged for 15 min at 10,000× *g* at 4 °C and the supernatant was collected. This procedure was repeated three times and supernatants from successive extractions were pooled. The extracts were then filtered through Whatman 1 filter paper and subsequently subjected to solvent evaporation in a Rotavapor (BUCHI R-100, Flawil, Switzerland), at <40 °C, to remove the organic solvent, and freeze-dried to remove the remaining water.

### 4.2. Experimental Diets

Four experimental diets were formulated to contain 48% protein and 18% lipids (Table 8). A practical plant-based diet was used as a control (N0F0) and three other diets were formulated similarly to the control, but were also supplemented with 1% NG (diet N1F0), 1% FV (diet N0F1), or 0.5% NG + 0.5% FV (diet N1F1). Diet designations denote presence (1)/absence (0) of the extracts. Dry ingredients were weighed and well mixed. To facilitate the inclusion of NG extract in the diets, the dry extract was dissolved in absolute ethanol, well mixed with the dry ingredients, and the mixture was dried in an oven at 40 °C until complete ethanol evaporation. Finally, oil and water were added. Diets were then pelleted (California Pellet Mill, Crawfordsville, IN, USA), dried in an oven at 40 °C for 48 h, and then stored in a freezer (−20 °C) until use. Composition and proximate analysis of the experimental diets is shown in Table 8.

### 4.3. Ethics Statement

Experiments were directed by accredited scientists, with category C status, following the Federation of European Laboratory Animal Science Associations (FELASA) recommendations and conducted according to the European Union directive 2010/63/EU on the protection of animals for scientific purposes.

### 4.4. Feeding Trial, Sampling and Acute Handling Stress Procedures

Meagre (*Argyrosomus regius*) juveniles were obtained from Instituto Português do Mar e da Atmosfera (IPMA), Olhão, Portugal, and acclimated for 3 weeks to the experimental conditions, during which they were fed a commercial diet with 44% protein and 18% lipids (Aquasoja Sustainable Feed, Sorgal, Ovar, Portugal). The trial was conducted at the Marine Zoology Station, Porto University, Portugal, in a thermo-regulated recirculating water system with 12 tanks of 100 L capacity, supplied with a continuous flow of filtered seawater (35 g L^−1^). A 12 h:12 h light: dark photoperiod was adopted, oxygen was maintained near saturation (7 mg L^−1^), and the temperature was set to 22 °C ± 1 °C. Thirteen fish with 28.8 ± 0.1 g average body weight were allocated in each tank and the diets were randomly assigned to triplicate tanks. Fish were hand-fed to apparent visual satiation twice a day, 6 days per week, for 4 weeks.

By the end of the feeding period, fish were fasted for 18 h before sampling and 3 fish were randomly sampled from each tank (*n* = 9) The remaining fish were bulk weighed under slight anaesthesia with ethylene glycol monophenyl ether (0.3:1000 v/v; Merck, Whitehouse Station, USA). Blood samples were immediately collected from the caudal vein using heparinised syringes and centrifuged for 10 min at 10,000× *g* to obtain plasma, which was stored at −20 °C until analyses. Fish were then euthanised by severing the nerve cord, weighed, and the liver and lateral muscle were collected, before being snap-frozen in liquid nitrogen and stored at −80 °C until oxidative status analyses were performed. Muscle and head-kidney were also collected, preserved overnight in RNA Later at 4 °C, and then stored at −80 °C until gene expression analyses were performed. These were henceforth designated the No Stress (NS) group.

The acute handling stress trial consisted of exposing the remaining fish to an acute handling stress through handling. For that purpose, fish were chased in the tanks for 1 min every 10 min for 1 h, and then caught with a nylon net and confined in a bucket for 10 min. Three fish were then sampled as described above and henceforth designated the acute handling stress (AS) group.

### 4.5. Chemical Analyses

The proximate composition of diets was analysed using the following procedures according to [75]: dry matter, after drying in an oven at 105 °C until constant weight; ash, by incineration in a muffle furnace at 450 °C for 16 h; crude protein (N × 6.25), by the Kjeldahl method, after acid digestion using a Kjeltec digestion and distillation system (Tecator Systems; models 1015 and 1026, respectively); lipids, by petroleum ether extraction in a SoxTec system (Tecator Systems; extraction unit model 1043 and service unit model 1046); gross energy content was determined using an oxygen bomb calorimeter (Parr 1281 Calorimeter, Parr Instrument Company, Moline, IL, USA).

### 4.6. Plasma Biochemistry

Commercial kits were used for the determination of cortisol (EIAHCOR, Invitrogen, CA, USA), lactate and glucose (Ref. 1001330 and 1001191, respectively; Spinreact, Girona, Spain). All plasmatic parameters were analysed using a colorimetric reaction, and absorbance was measured in a Multiskan GO microplate reader (Model 5111 9200; Thermo Scientific, Nanjing, China).

### 4.7. Hematological Analysis

Fresh heparinised blood was used for hematocrit (Ht) determination and blood cell counts. Ht, total red blood cells (RBC), white blood cells (WBC), and differential WBC counts were determined as described by [76].

### 4.8. Gene Expression Analysis

Total RNA was extracted from samples of muscle and head kidney, using the Direct-zol™ RNA MiniPrep Kit (Zymo Research, Irvine, CA, USA). Samples were homogenised in 600 μL TRI Reagent using the Precellys 24 homogeniser (Bertin Technologies, Montigny-Le-Bretonneux, France), in a 2 mL vial and then centrifuged at 13,000× *g* for 1 min at 4 °C. The supernatant was collected and mixed with an equal volume of absolute ethanol (PanReac, Barcelona, Spain). The mixture was transferred into a spin column (supplied with the kit) and centrifuged for 1 min at 13,000× *g*, at 4 °C. The RNA was then washed and subjected to DNase treatment, following the kit manufacturer’s instructions. Finally, RNA was eluted in 50 µL of DEPC-treated water and subjected to electrophoresis on 1% agarose gels to confirm the integrity of the 28S and 18S rRNA bands. RNA quality was measured using the μDrop™ Plate (Thermo Scientific, Courtaboeuf, France) in a Multiskan GO Spectrophotometer (Thermo Scientific, Courtaboeuf, France).

cDNA was generated from 1 µg of total RNA using the NZY First-Strand cDNA Synthesis Kit (NZYTech, Lisbon, Portugal), following the manufacturer’s protocol. The product of the first strand cDNA synthesis was stored at −20 °C until further analysis.

Gene expression of heat shock proteins 70 (HSP70) and 90 (HSP90) in the muscle and the immune-related proinflammatory cytokines, interleukine-1-beta (IL1-β) and tumour necrosis factor-alpha (TNF-α), and the anti-inflammatory cytokine, interleukine-10 (IL-10), in the head-kidney were determined using real-time quantitative PCR (CFX Connect™ Real-Time System, Bio-Rad, Hercules, CA, USA). cDNA amplification was performed using specific PCR primers, retrieved from [77] and designed in the present work (Table 9). To assess HSP gene expression, and since no nucleotide sequences were available in the literature for meagre, identification and primer design were performed for the genes of interest. First, the nucleotide sequences of genes of interest from species that are closely related to meagre were blasted using the NCBI database. To identify conserved regions, the nucleotide sequence was aligned using MAFFT alignment software (https://www.ebi.ac.uk/Tools/msa/mafft/ accessed in 1 January 2020). Then, based on the open reading frame (ORF), primers were designed on the identified conserved regions (primer length 20–23 bp; product size 100–250 bp; Tm 60 ± 1 °C; G/C ≤ 50%) and their quality determined using the ThermoFisher Scientific Multiple Primer Analyser software (https://www.thermofisher.com/pt/en/home/brands/thermo-scientific/molecular-biology/molecular-biology-learning-center/molecular-biology-resource-library/thermo-scientific-web-tools/multiple-primer-analyzer.html; accessed in 1 January 2020). To determine primer efficiency, seven serial two-fold dilutions of a cDNA mix of all samples were prepared, and efficiency was calculated from the slope of the regression line of the quantification cycle (Ct) versus the log 10 of the cDNA different dilutions [78]. Real-time qPCR reactions were performed using 3.5 μL of ultrapure water (Sigma-Aldrich, Taufkirchen, Germany), 5 μL of SsoAdvanced Universal SYBR^®^ Green supermix (Bio-Rad, Hercules, CA, USA), 0.5 μL of each primer and 1 μL cDNA from each sample, adding up to a final volume reaction of 10 μL. The different transcripts were amplified using technical triplicates per sample, under the following conditions: 95 °C for 30 s for denaturation, followed by 40 cycles of 95 °C for 15 s, and 58 °C for 30 s. A melting curve analysis was performed to verify that only specific amplification occurred, and no primer dimers were amplified. The relative expression of each transcript was normalised to the selected housekeeping gene (elongation-factor 1-alpha, EF-1α), due to its expression stability in the intestine and calculated using the Pfaffl method [78].

### 4.9. Enzyme Activity

Liver and muscle samples were diluted to 1:9 and 1:3, respectively, and homogenised in ice-cold 100 mM Tris–HCl buffer containing 0.1 mM EDTA and 0.1% (v/v) Triton X-100 at pH 7.8, using Precellys^®^ Evolution homogeniser (Bertin Corp., Rockville, MD, USA). Homogenates were centrifuged at 30,000× *g* for 30 min at 4 °C and the resultant supernatants were separated in aliquots and stored at −80 °C for further enzyme assays.

Superoxide dismutase (SOD, EC 1.15.1.1), catalase (CAT, EC 1.11.1.6), glutathione peroxidase (GPX, EC 1.11.1.9), glutathione reductase (GR, EC 1.6.4.2), and glucose 6-phosphate dehydrogenase (G6PDH, EC 1.1.1.49) activities were determined in liver and muscle, as described by [76]. Protein concentration in homogenates was determined by the Bradford method [79] using BioRad Protein Assay Dye Reagent (Ref. 5000006) with bovine albumin as standard. All enzymatic activity analyses were carried out at 37°C. A Multiskan GO microplate reader (Model 5111 9200; Thermo Scientific, Nanjing, China) was used to monitor the changes in absorbance.

For SOD, one unit of enzyme activity was defined as the amount of enzyme necessary to produce 50% inhibition of the ferricytochrome C reduction rate. All other enzyme activities were expressed as units (CAT) or milliunits (G6PDH, GPX, and GR) per milligram of hepatic soluble protein (specific activity). One unit of enzyme activity was defined as the amount of enzyme required to transform 1 μmol of substrate per minute under the assay conditions.

### 4.10. Lipid Peroxidation (LPO)

Malondialdehyde (MDA) concentration was used as a marker of LPO levels in the liver and muscle. In the presence of thiobarbituric acid, MDA reacts producing coloured thiobarbituric acid-reacting substances (TBARS) that were measured as described in [76] The results were expressed as nanomoles MDA per gram of wet tissue, calculated from a calibration curve.

### 4.11. Total and Oxidised Glutathione (tGSH and GSSG)

Liver and muscle samples were homogenised (1:9 and 1:3, respectively) in an ice-cold solution of 1.3% 5-sulfosalicylic acid (*w/v*) and 10 mM HCl using Precellys 24 homogeniser (Bertin Technologies). All procedures were carried out on ice to avoid glutathione oxidation. Homogenates were centrifuged at 14,000× *g* for 10 min at 4 °C and the resulting supernatants stored at −80 °C. Total glutathione (tGSH) and oxidised glutathione (GSSG) were measured as described by [80]. Standard curves of reduced glutathione (GSH) and GSSG were used for tGSH and GSSG calculations, respectively. GSH levels were calculated by subtracting GSSG from tGSH values. The oxidative stress index (OSI) was calculated through the following equation:

OSI = 100 × (2 × GSSG)/tGSH(1)

### 4.12. Statistical Analysis

Before analyses, data were tested for normality and homogeneity using Shapiro–Wilk and Levene tests, respectively. The experimental unit considered was the fish (*n* = 9). Growth and feed utilization was analysed by a two-way Analysis of Variance (ANOVA), with *N. gaditana* and *F. vesiculosus* as factors. Data on plasma biochemistry, haematology and oxidative stress were analysed by three-way ANOVA with *N. gaditana*, *F. vesiculosus*, and Stress as factors. When significant interactions between two factors were observed, data were analysed by one-way ANOVA to disclose each factor’s main effects. Plasma cortisol and gene expression data were not normal nor homogeneous and could not be normalised. Thus, differences between different diets for each condition (AS or NS) were analysed using the Kruskal–Wallis test followed by multiple pairwise comparison. Within each dietary treatment (N0F0, N0F1, N1F0 and N1F1), differences between AS and NS groups were assessed using the Mann–Whitney U non-parametric test. Differences were considered statistically significant at *p* < 0.05. All statistical analyses were performed using the IBM SPSS Statistics v25 software (IBM Corp.).

## Figures and Tables

**Table 1:** Growth performance, ingestion and feed efficiency of meagre (initial body weight (IBW)—28.8 g) fed the experimental diets for four weeks.

Diets	Final Body Weight (g)	Weight Gain (%IBW)	Feed Intake (g kg ABW—1; day—1)	Feed Efficiency
N0F0	42.7 ± 1.17	48 ± 5	8.0 ± 0.7	0.6 ± 0.1
N0F1	45.9 ± 3.22	59 ± 14	8.3 ± 1.1	0.7 ± 0.1
N1F0	41.9± 3.39	40 ± 14	9.1 ± 1.2	0.5 ± 0.1
N1F1	40.3 ± 2.15	45 ± 9	8.1 ± 1.0	0.6 ± 0.0
Two-way ANOVA ^†^	Final Body Weight	Feed Intake	Feed Intake	Feed Efficiency
N	ns	ns	ns	*
F	ns	ns	ns	*
N * F	ns	ns	ns	ns

ABW—Average body weight = (initial body weight + final body weight)/2. FE—Feed efficiency = weight gain/dry feed intake. ^†^ Data are presented as mean ± SD (*n* = 3). Data were analysed by a 2-way ANOVA with *Nannochloropsis* (N) and *Fucus* (F) as factors; ns: non-significant; * *p* < 0.05.

**Table 2:** Plasma biochemistry parameters for no-stress and acute-handling-stress meagre fed the experimental diets.

Treatment	Diets	Glucose (mmol L^−1^)		Lactate (mmol L^−1^)		Cortisol (μg L^−1^)	
No stress (NS)	N0F0	3.7 ± 0.9		3.9 ± 1.2		1.6 ± 0.0	
	N0F1	3.3 ± 0.7		3.4 ± 1.3		1.0 ± 0.4	
	N1F0	3.0 ± 0.6		3.5 ± 0.8		0.9 ± 0.5	
	N1F1	3.0 ± 0.6		3.8 ± 0.9		1.4 ± 1.4	
Acute handling stress (AS)	N0F0	5.1 ± 0.5		3.5 ± 0.9		6.2 ± 3.5	
	N0F1	4.6 ± 0.9		2.7 ± 1.1		12.5 ± 7.2	
	N1F0	4.9 ± 0.8		2.6 ± 0.9		15.5± 21.8	
	N1F1	4.8 ± 0.7		2.9 ± 0.8		14.6 ± 4.8	
Three-way ANOVA ^†^						Mann–Whitney	
		Glucose		Lactate		Cortisol	
N		*		ns		ns	
F		*		ns		ns	
Stress		***		***		***	
N * F		ns		*		-	
N * Stress		*		ns		-	
F * Stress		ns		ns		-	
N * F * Stress		ns		ns		-	
Glucose Interactions		No stress	Acute stress	N0	N1	F0	F1
N0 vs. N1		**	ns	-	-	-	-
AS vs. NS		-	-	***	***	-	-
Lactate Interactions							
F0 vs. F1		-	-	*	ns	-	-
N0 vs. N1		-	-	-	-	*	ns

^†^ Data are presented as mean ± SD (*n* = 9). Data were analysed by a 3-way ANOVA with *N. gaditana* (N), *F. vesiculosus* (F) and Stress as factors. When interaction between 2 factors was significant (*p* < 0.05), analysis of the main effects for each factor was conducted by one-way ANOVA. Cortisol was analysed using the non-parametric Mann–Whitney U test; ns: non-significant; * *p* < 0.05; ** *p* < 0.01; *** *p* < 0.001.

**Table 3:** Hematological profile of no-stress and acute-handling-stress meagre fed the experimental diets.

Treatment	Diets	RBC (10^6^ mm^−3^)	WBC (10^4^ mm^−3^)	Thrombocytes (10^4^ mm^−3^)	Lymphocytes (10^4^ mm^−3^)	Monocytes (10^4^ mm^−3^)	Neutrophils (10^4^ mm^−3^)	Hematocrit (%)	MCV
No stress (NS)	N0F0	1.5 ± 0.3	7.8 ± 0.9	115.0 ± 7.0	74.8 ± 6.4	7.7 ± 5.0	2.6 ± 3.3	22.1 ± 2.6	153.2 ± 18.2
	N0F1	1.4 ± 0.2	7.8 ± 1.0	118.2 ± 6.7	74.2 ± 6.0	5.9 ± 4.2	1.7 ± 1.9	21.1 ± 5.0	155.4 ± 64.2
	N1F0	1.4 ± 0.3	7.2 ± 1.3	122.0 ± 5.1	73.7 ± 5.2	3.8 ± 2.2	0.6 ± 0.5	19.8 ± 2.7	144.8 ± 26
	N1F1	1.4 ± 0.2	8.6 ± 1.4	115.1 ± 6.2	76.9 ± 4.8	5.8 ± 2.4	2.2 ± 2.7	19.9 ± 0.8	142.8 ± 18.0
Acute handling stress (AS)	N0F0	1.5 ± 0.3	7.0 ± 2.5	121.3 ± 8.6	66.8 ± 7.5	8.6 ± 4.4	3.3 ± 3.4	23.7 ± 2.5	161.2 ± 34.2
	N0F1	1.5 ± 0.1	7.9 ± 1.0	121.8 ± 5.0	67.8 ± 4.4	8.6 ± 4.6	1.9 ± 1.8	19.6 ± 2.4	131.4 ± 18.2
	N1F0	1.6 ± 0.2	7.4 ± 1.1	120.8 ± 11.4	67.4 ± 8.1	9.8 ± 4.2	2.7 ± 1.6	21.2 ± 2.0	135.7 ± 20.2
	N1F1	1.6 ± 0.2	8.2 ± 0.9	122.2 ± 8.2	67.4 ± 7.5	4.3 ± 3.0	6.0 ± 4.3	23.2 ± 2.8	144.3 ± 23.1
Three-way ANOVA ^†^		RBC (10^6^ mm^−3^)	WBC (10^4^ mm^−3^)	Thrombocytes	Lymphocytes	Monocytes	Neutrophils	Hematocrit (%)	MCV
N		ns	ns	ns	ns	ns	ns	ns	ns
F		ns	*	ns	ns	ns	ns	ns	ns
Stress		*	ns	*	***	*	**	ns	ns
N * F		ns	ns	ns	ns	ns	**	ns	ns
N * Stress		ns	ns	ns	ns	ns	ns	*	ns
F * Stress		ns	ns	ns	ns	ns	ns	ns	ns
N * F * Stress		ns	ns	ns	ns	ns	ns	ns	ns
Neutrophils Interactions			No stress	Acute stress	N0	N1	F0		F1
F0 vs. F1			-	-	ns	*	-		-
N0 vs. N1			-	-	-	-	ns		*
Hematocrit Interactions									
AS vs. NS			-	-	ns	*	-		-
N0 vs. N1			*	ns	-	-	-		-

RBC—Total red blood cells; WBC—white blood cells; MCV—mean corpuscular volume. When interaction between 2 factors was significant (*p* < 0.05), analysis of the main effects for each factor was conducted by one-way ANOVA. Cortisol was analysed using the non-parametric Mann–Whitney U test; ns: non-significant; * *p* < 0.05; ** *p* < 0.01; *** *p* < 0.001.

**Table 4:** Hepatic antioxidant enzymes activity in no-stress and acute-handling-stress meagre fed the experimental diets.

Treatment	Diets	SOD	CAT	GPX	GR	G6PDH
No stress (NS)	N0F0	444.5 ± 52.1	318.5 ± 75.8	135.7 ± 24.2	13.3 ± 0.8	73.4 ± 18.8
	N0F1	462.3 ± 77.3	358.2 ± 57.9	156.1 ± 25.3	12.6 ± 1.3	87.5 ± 13.3
	N1F0	334.9 ± 40.0	317.1 ± 54.2	136.5 ± 23.8	12.3 ± 1.5	75.3 ± 19.5
	N1F1	395.2 ± 52.8	327.7 ± 53.8	138.3 ± 26.9	11.6 ± 1.4	73.1 ± 11.7
Acute handling stress (AS)	N0F0	622.1 ± 134.7	308.1 ± 46.2	626.9 ± 100.8	15.1 ± 0.6	98.3 ± 10.0
	N0F1	576.9 ± 149.2	300.2 ± 33.7	591.8 ± 89.1	13.6 ± 2.3	88.9 ± 20.7
	N1F0	538.4 ± 125.2	287.4 ± 36.1	574.7 ± 56.1	13.6 ± 0.8	78.3 ± 13.3
	N1F1	401.9 ± 53.0	321 ± 24.7	595.4 ± 56.1	13.6 ± 2.2	85.6 ± 15.6
Three-way ANOVA ^†^		SOD	CAT	GPX	GR	G6PDH
N		***	ns	ns	*	*
F		ns	ns	ns	ns	ns
Stress		***	ns	***	***	*
N * F		ns	ns	ns	ns	ns
N * Stress		ns	ns	ns	ns	ns
F * Stress		*	ns	ns	ns	ns
N * F * Stress		ns	ns	ns	ns	ns

SOD—Superoxide distumase (U mg protein^−1^); CAT—Catalase (U mg protein^−1^); GPX—Gluthatione Peroxidase; GR—Gluthatione Reductase (mU mg protein^−1^); G6PDH—Glucose-6-Phosphate Dehydrogenase (mU mg protein^−1^). ^†^ Data are presented as mean ± SD (*n* = 9). Data were analysed by a 3-way ANOVA with *N. gaditana* (N), *F. vesiculosus* (F) and Stress as factors. When interaction between 2 factors was *p* < 0.05, analysis of the main effects for each factor was conducted via a one-way ANOVA; ns: non-significant; * *p* < 0.05; *** *p* < 0.001.

**Table 5:** Hepatic lipid peroxidation, glutathione (total, reduced and oxidised glutathione), and oxidative stress index of no-stress and acute-stress meagre fed the experimental diets.

Treatment	Diets	tGSH	GSH	GSSG	OSI	LPO
No stress (NS)	N0F0	1924.5 ± 265.5	1819.7 ± 282.1	104.8 ± 58.4	11.2 ± 6.7	21.8 ± 6.4
	N0F1	2015 ± 309.5	1887 ± 314	86.7 ± 22.9	8.8 ± 3.1	24.1 ± 12.8
	N1F0	2030.4 ± 92.3	1951.9 ± 123.3	78.5 ± 42.2	7.9 ± 4.6	20.3 ± 4.5
	N1F1	1896.8 ± 270.3	1825.6 ± 271.7	71.2 ± 32.6	7.6 ± 3.6	22.6 ± 9
Acute stress (AS)	N0F0	1817.4 ± 117.3	1747.2 ± 123.9	70.2 ± 38.2	7.8 ± 4.1	25.2 ± 3.6
	N0F1	1856.6 ± 209.4	1772.4 ± 240.6	71.8 ± 21.3	7.6 ± 2.6	29.3 ± 7.2
	N1F0	1624.1 ± 136.4	1599.4 ± 291	91.6 ± 46.3	11.1 ± 5.5	27.8 ± 8.9
	N1F1	1573.7 ± 110.3	1531.4 ± 109.4	44.2 ± 21	5.4 ± 2.7	21.7 ± 5.1
Three-way ANOVA ^†^		tGSH	GSH	GSSG	OSI	LPO
N		*	ns	ns	ns	ns
F		ns	ns	ns	ns	ns
Stress		***	**	ns	ns	ns
N * F		ns	ns	ns	ns	ns
N * Stress		*	ns	ns	ns	ns
F * Stress		ns	ns	ns	ns	ns
N * F *Stress		ns	ns	ns	ns	ns
tGSH Interactions			No stress	Acute stress	N0	N1
N0 vs. N1			ns	**	-	-
AS vs. NS			-	-	ns	***

LPO—lipid peroxidation (nmol malondialdehyde. g tissue^−1^); tGSH—total glutathione; GSH—reduced glutathione (nmol/g tissue); GSSG—oxidised glutathione (nmol/ g tissue); OSI—oxidative stress index. ^†^ Data are presented as mean ± SD (*n* = 9). Data were analysed by a 3-way ANOVA with N. gaditana (N), F. vesiculosus (F) and Stress as factors. When interaction between 2 factors was *p* < 0.05, analysis of the main effects for each factor was conducted via a one-way ANOVA; ns: non-significant; * *p* < 0.05; ** *p* < 0.01; *** *p* < 0.001.

**Table 6:** Muscle antioxidant enzymes activity in no-stress and acute-handling-stress meagre fed the experimental diets.

Treatment	Diets	SOD	CAT	GPX	GR	G6PDH	
No stress (NS)	N0F0	101.8 ± 19.7	0.9 ± 0.2	18.9 ± 1.8	1.4 ± 0.5	0.5 ± 0.1	
	N0F1	106.8 ± 32.8	0.9 ± 0.2	23.4 ± 3.8	1.9 ± 0.3	0.6 ± 0.2	
	N1F0	114.3 ± 13.1	1.0 ± 0.2	20.8 ± 4.4	1.6 ± 0.3	0.4 ± 0.1	
	N1F1	88.1 ± 24.8	1.1 ± 0.3	22.0 ± 3.5	1.9 ± 0.5	0.5 ± 0.2	
Acute stress (S)	N0F0	101.8 ± 31.7	1.3 ± 0.4	18.1 ± 3.8	1.5 ± 0.3	0.5 ± 0.2	
	N0F1	116.5 ± 40.3	1.3 ± 0.6	20.8 ± 4.7	1.8 ± 0.4	0.6 ± 0.2	
	N1F0	107.5 ± 18.0	1.5 ± 0.4	16.0 ± 6.7	1.6 ± 0.4	0.5 ± 0.1	
	N1F1	103.1 ± 24.4	1.3 ± 0.4	15.1 ± 3.8	1.3 ± 0.2	0.5 ± 0.1	
Three-way ANOVA ^†^		SOD	CAT	GPX	GR	G6PDH	
N		ns	*	ns	ns	*	
F		ns	ns	ns	*	ns	
Stress		ns	*	**	ns	ns	
N * F		ns	ns	ns	*	ns	
N * Stress		ns	ns	ns	ns	ns	
F * Stress		ns	ns	ns	*	ns	
N * F * Stress		ns	ns	ns	ns	ns	
GR Interactions		No stress	Acute stress	N0	N1	F0	F1
F0 vs. F1		*	ns	*	ns	-	-
NS vs. AS		-	-	-	-	ns	*
N0 vs. N1		-	-	-	-	ns	*

SOD—Superoxide distumase (U mg protein^−1^); CAT—Catalase (U mg protein^−1^); GPX—Gluthatione Peroxidase; GR—Gluthatione Reductase (mU mg protein^−1^); G6PDH—Glucose-6-Phosphate Dehydrogenase (mU mg protein^−1^). ^†^ Data are presented as mean ± SD (*n* = 9). Data were analysed by a 3-way ANOVA with N. gaditana (N), F. vesiculosus (F) and Stress as factors. When interaction between 2 factors was *p* < 0.05, analysis of the main effects for each factor was conducted via a one-way ANOVA; ns: non-significant; * *p* < 0.05; ** *p* < 0.01.

**Table 7:** Muscle lipid peroxidation, glutathione (total, reduced and oxidised glutathione), and oxidative stress index of no-stress and acute-handling-stress meagre fed the experimental diets.

Treatment	Diets	tGSH	GSH	GSSG	OSI	LPO
No stress (NS)	N0F0	179.3 ± 22.5	174.9± 22.0	5.1 ± 0.7	5.6 ± 0.9	1.7 ± 1.1
	N0F1	189.4 ± 26.1	184.5 ± 26.3	4.6 ± 1.2	5.2 ± 1.6	0.6 ± 0.4
	N1F0	165.1 ± 22.1	160.4 ± 23.5	4.7 ± 1.6	6.1 ± 2.9	0.9 ± 0.6
	N1F1	172.0 ± 35.1	164.8 ± 34.8	7.0 ± 5.3	6.3 ± 2.1	0.6 ± 0.4
Acute stress (S)	N0F0	194.9 ± 24.2	186.3± 24.3	8.6 ± 4.8	8.9 ± 5.1	2.3 ± 1.1
	N0F1	188.9± 32.1	179.8± 31.5	9.1 ± 3.9	9.9 ± 4.5	1.5 ± 0.4
	N1F0	200.7 ± 23.5	190.3 ± 22.6	9.8 ± 4.5	10.3 ± 4.2	1.7 ± 0.5
	N1F1	184.6 ± 23.3	173.1 ± 25.5	11.5 ± 3.5	13.0 ± 5.1	2.2 ± 1.7
Three-way ANOVA ^†^		tGSH	GSH	GSSG	OSI	LPO
N		ns	ns	ns	ns	ns
F		ns	ns	ns	ns	*
Stress		*	ns	***	***	***
N * F		ns	ns	ns	ns	ns
N * Stress		ns	ns	ns	ns	ns
F * Stress		ns	ns	ns	ns	ns
N * F * Stress		ns	ns	ns	ns	ns

LPO—lipid peroxidation (nmol malondialdehyde. g tissue-1); tGSH—total glutathione; GSH—reduced glutathione (nmol/g tissue); GSSG—oxidised glutathione (nmol/g tissue); OSI—oxidative stress index. ^†^ Data are presented as mean ± SD (*n* = 9). Data were analysed by a 3-way ANOVA with N. gaditana (N), F. vesiculosus (F) and Stress as factors; ns: non-significant; * *p* < 0.05; *** *p* < 0.001.

**Table 8:** Composition and proximate analysis of the experimental diets.

	Diets			
	N0F0	N1F0	N0F1	N1F1
**Ingredients (% dry weight)**				
Fish meal ^1^	25.0	25.0	25.0	25.0
Wheat gluten ^2^	5.0	5.0	5.0	5.0
Corn gluten ^3^	15.0	15.0	15.0	15.0
Soybean meal ^4^	32.4	32.4	32.4	32.4
Wheat meal ^5^	0.9	0.9	0.9	0.9
Cellulose	1.5	0.5	0.5	0.5
Ammonium phosphate	1.3	1.3	1.3	1.3
Cod liver oil	5.2	5.2	5.2	5.2
Soy bean oil	5.0	5.0	5.0	5.0
Rapeseed oil	5.0	5.0	5.0	5.0
Taurine ^6^	0.2	0.2	0.2	0.2
Vitamin mix ^7^	1.0	1.0	1.0	1.0
Mineral mix ^8^	1.0	1.0	1.0	1.0
Binder ^9^	1.0	1.0	1.0	1.0
Choline chloride (50%)	0.5	0.5	0.5	0.5
*N. gaditana* extract ^10^	0.0	1.0	0.0	0.5
*F. vesiculosus* extract ^11^	0.0	0.0	1.0	0.5
**Proximate analyses (% dry weight)**				
Dry matter (%)	92.4	93.6	92.7	93.7
Crude protein (%)	48.1	48.4	49.6	49.7
Crude fat (%)	15.2	16.0	15.7	16.0
Gross Energy (kJ g^−1^ % DM)	22.2	22.3	22.4	22.4
Ash (%)	11.4	11.3	11.6	11.4

CP—Crude Protein. TL—Total lipid. ^1^ Sorgal, S.A. Ovar, Portugal (CP: 72.4%; TL: 17.0%). ^2^ Sorgal, S.A. Ovar, Portugal (CP: 84.6%; TL: 1.0%).^3^ Sorgal, S.A. Ovar, Portugal (CP: 69.9%; TL: 3.3%) ^4^ Sorgal, S.A. Ovar, Portugal (CP: 54.2%; TL: 1.8%). ^5^ Sorgal, S.A. Ovar, Portugal (CP: 13.8%; TL: 1.1%). ^6^ Feed-grade, Sorgal, S.A. Ovar, Portugal. ^7^ Vitamins (mg kg^−1^ diet): retinol, 18,000 (IU kg^−1^ diet); calciferol, 2000 (IU kg^−1^ diet); alpha tocopherol, 35; menadion sodium bis., 10; thiamin, 15; riboflavin, 25; calcium pantothenate, 50; nicotinic acid, 200; pyridoxine, 5; folic acid, 10; cyanocobalamin, 0.02; biotin, 1.5; ascorbyl monophosphate, 50; inositol, 400. ^8^ Minerals (mg kg^−1^ diet): cobalt sulphate, 1.91; copper sulphate, 19.6; iron sulphate, 200; sodium fluoride, 2.21; potassium iodide, 0.78; magnesium oxide, 830; manganese oxide, 26; sodium selenite, 0.66; zinc oxide, 37.5; dicalcium phosphate, 8.02 (g kg^−1^ diet); potassium chloride, 1.15 (g kg^−1^ diet); sodium chloride, 0.4 (g kg^−1^ diet). ^9^ Aquacube. Agil, UK. ^10^ Total phenolic content (TPC): 0.72 ± 0.08 mg galic acid (GA) g^−1^ dry weight; 2,2’-azino-bis(3-ethylbenzothiazoline-6-sulfonic acid (ABTS) radical scavenging activity: 24.97 ± 2.01 μmol 6-hydroxy-2,5,7,8-tetramethylchroman-2-carboxylic acid (Trolox) g^−1^ dry weight; 2,2-diphenyl-1-picrylhydrazyl (DPPH) radical scavenging activity: 5.78 ± 0.48 μmol Trolox g^−1^ dry weight. (Determined in [26].). ^11^ Total phenolic content (TPC): 7.93 ± 0.34 mg GA g^−1^ dry weight; ABTS radical scavenging activity: 99.37 ± 11.27 μmol Trolox g^−1^ dry weight; DPPH radical scavenging activity: 81.54 ± 6.71 μmol Trolox g^−1^ dry weight. (Determined in [26].).

**Table 9:** Primer sequences used for transcript amplification by RT-PCR.

Gene	Sequence	Efficiency	Amplicon (bp)	Reference
EF-1α	F: TACGGTTCCGATACCGCCG	2.0	189	[77]
	R: AACATGCTTGAGGGCAGTGACAA			
IL-1β	F: GATTGCCTGGATTTTCCACTGTCTCCA	2.3	103	[77]
	R: GTGGCTCTGGGCATCAAGGG			
IL-10	F: ACTCCTCGGTCTCTCCTCGTATCCGC	2.1	187	[77]
	R: CTGTGTCGAGATCATCGTTGGCTTCATAAAAGTC			
TNF-α	F: CACAAGAGCGGCCATTCATTTACAAGGAG	1.8	173	[77]
	R: GGAAAGACGCTTGGCTGTAGATGG			
HSP70	F: ATCACAGTTCCGGCGTATTT	1.9	197	Present study
	R: ATGGACACGTCAAAGGTGCC			
HSP90	F: ATCGTGGAGACTCTCAGGCA	1.9	146	Present study
	R: CTGTAGATGCGGTTGGAGTG			

## Data Availability

The data presented in this study are available on request from the corresponding author.
